# Crystal structure of Ba_2_Co(BO_3_)_2_

**DOI:** 10.1107/S2056989019002597

**Published:** 2019-02-22

**Authors:** Fatima-Ezahra N’Faoui, Jilali Aride, Ali Boukhari, M’Hamed Taibi, Mohamed Saadi, Lahcen El Ammari

**Affiliations:** a Laboratoire de Physico-Chimie des Matériaux Inorganiques et Organiques, Centre des Sciences des Matériaux, Ecole Normale Supérieure, Mohammed V University in Rabat, Morocco; b Laboratoire de Chimie Appliquée des Matŕiaux, Centre des Sciences des Matériaux, Faculty of Sciences, Mohammed V University in Rabat, Avenue Ibn Batouta, BP 1014, Rabat, Morocco

**Keywords:** cobalt borate, diborates, Ba_2_Co(BO_3_)_2_, crystal structure, crystal growth, X-ray diffraction

## Abstract

Single crystals of dibarium cobalt(II) bis­(orthoborate), Ba_2_Co(BO_3_)_2_, have been obtained from the melt. The crystal structure is composed of two isolated (BO_3_)^3−^ triangles linked to Co^2+^ cations. The resulting [CoO_5_] square pyramids and the borate anions make up branched rows extending parallel to [010]. The barium cations occupy two sites in the voids of this arrangement and exhibit coordination numbers of nine each. A comparison with the structures of other *A*_2_ *M*(BO_3_)_2_ compounds reveals a unique five-coordination of the small metal *M* in the title compound instead of four- or six-coordination for the other *A*_2_ *M*(BO_3_)_2_ compounds with *M* = Cu, Zn, Mg, Ca, or Cd.

## Abstract 2

Ba_2_Co(BO_3_)_2_ crystallizes in a novel structure type, with [CoO_5_] square pyramids linked to BO_3_ triangles through corner- or edge-sharing, and the Ba^2+^ cations in the voids of this arrangement.

## Chemical context   

The crystal chemistry of borates differs from those of silicates, phosphates, sulfates, carbonates or nitrates due to the possibility of forming borate anions with trigonal–planar and tetra­hedral configurations (Zhang *et al.*, 2011*a* ▸; Filatov & Bubnova, 2000 ▸; Chen *et al.*, 2005 ▸; Reshak, 2016 ▸). In general, borate compounds are applied in different fields, such as non-linear optical (NLO) materials (Becker, 1998 ▸), for photoluminescence (Mergen & Pekgözlü, 2013 ▸), for their optical properties (Zhang *et al.*, 2011*b* ▸; Lv *et al.*, 2018 ▸), or as ferroelectrics (Dhanasekaran, 2009 ▸; Murugan *et al.*, 2001 ▸). The borate systems *A*_2_ *M*(BO_3_)_2_, where *A* = Ba, Sr, Pb and *M* = Cu, Mg, Cd, Ca, Zn, have been studied previously. For these compounds, several structures types have been reported that depend on the size and nature of the *A* and *M* atoms, as shown in Table 1 ▸.

In this investigation we have isolated single crystals of Ba_2_Co(BO_3_)_2_ from the melt. The new compound crystallizes in the monoclinic system in the same space-group type as some other *A*_2_ *M*(BO_3_)_2_ compounds, but with different cell parameters (Table 1 ▸).

## Structural commentary   

In the crystal structure of the title compound, except for the two oxygen atoms O1 and O3 that lie in general positions of the *C*2/*m* space group, all atoms are located on a mirror plane (Wyckoff position 4*i*).

**Figure 1:**
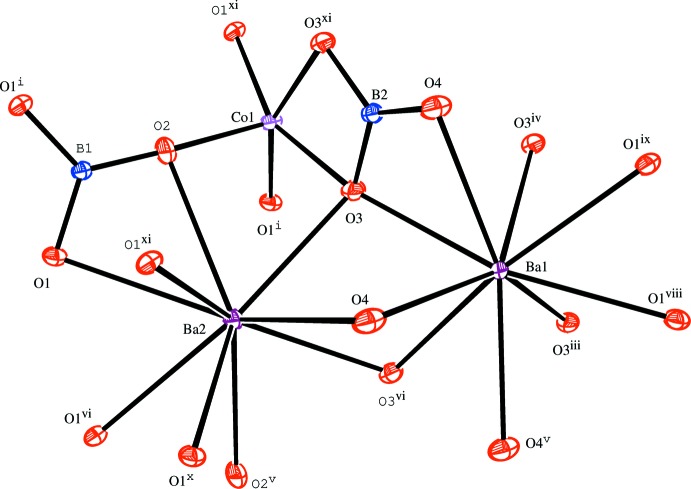
The principal building units in the structure of the title compound with the atom-labelling scheme. Displacement ellipsoids are drawn at the 50% probability level. [Symmetry codes: (i) *x*, −*y*, *z*; (ii) *x*, *y* − 1, *z*; (iii) −*x* + 1, −*y*, −*z* + 1; (iv) −*x* + 1, *y*, −*z* + 1; (v) −*x* + 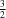, −*y* + 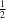, −*z* + 1; (vi) *x* + 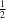, −*y* + 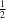, *z*; (vii) *x* + 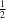, *y* − 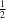, *z*; (viii) −*x* + 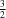, −*y* + 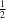, −*z* + 2; (ix) −*x* + 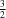, −*y* − 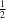, −*z* + 2; (*x*) −*x* + 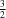, *y* − 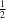, −*z* + 2; (xi) *x*, −*y* + 1, *z*.]

**Figure 2:**
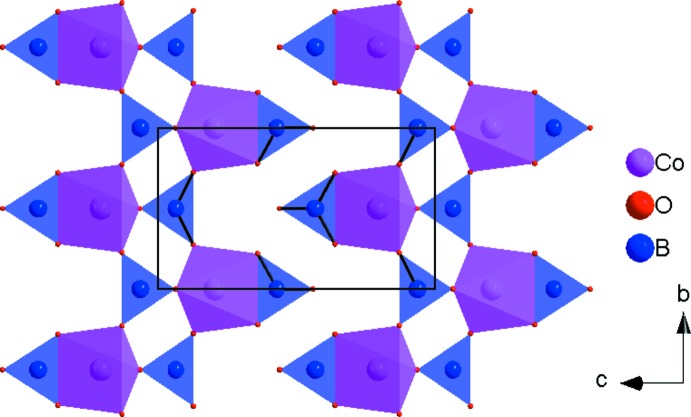
Edge- and corner-sharing [CoO_5_] and (BO_3_) polyhedra forming branched rows parallel to [010].

**Figure 3:**
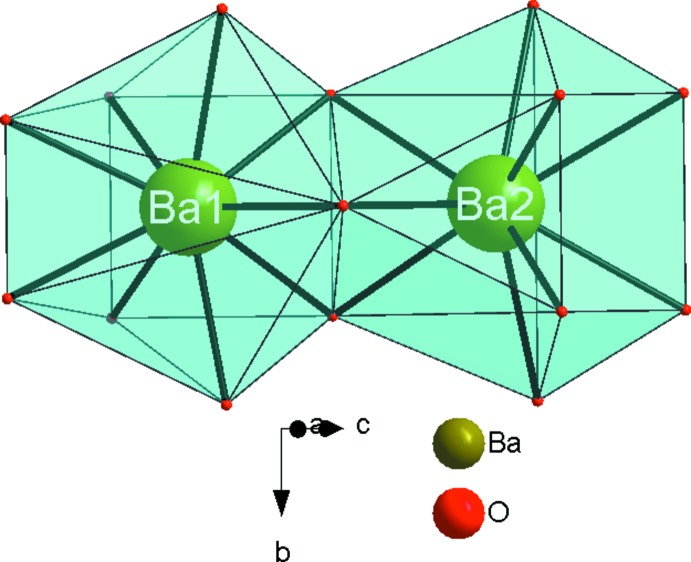
A pair of [BaO_9_] polyhedra.

**Figure 4:**
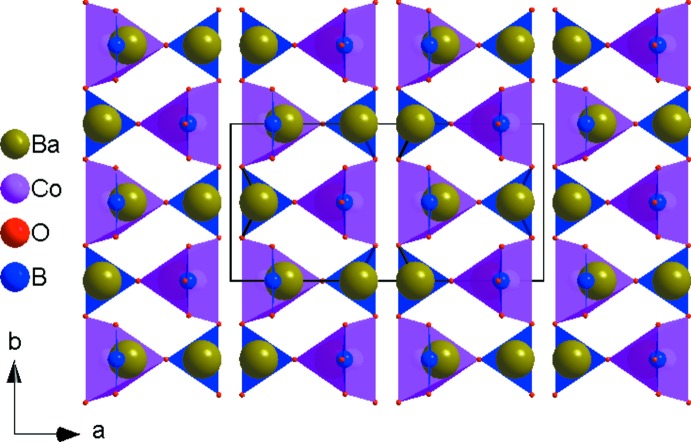
Projection of the crystal structure of Ba_2_Co(BO_3_)_2_ along [001].

The principal building units in the crystal structure are two trigonal–planar borate anions, one five-coordinate Co^2+^ cation and two nine-coordinate Ba^2+^ cations (Fig. 1 ▸). Relevant bond lengths and angles are collated in Table 2 ▸. The borate anions are isolated from each other. (B2O_3_) anions and [CoO_5_] polyhedra share one edge to form a {BCoO_6_} group, whereas the (B1O_3_) anion is connected through its corners to three different {BCoO_6_} groups. This arrangement leads to the formation of branched rows extending parallel to [010], as shown in Fig. 2 ▸. The rows are linked by pairs of [BaO_9_] polyhedra (Fig. 3 ▸) into a three-dimensional framework, as shown in Figs. 4 ▸ and 5 ▸.

The slight deviation of the boron atoms from a planar environment by oxygen atoms is reflected in the maximum deviation of 0.007 (3) Å for B1, compared with 0.019 (3) Å for B2. The average distances B1—O = 1.384 Å and B2—O = 1.387 Å are similar to those found in other *A*_2_ *M*(BO_3_)_2_ borates where B—O bonds vary between 1.325 and 1.411 Å and are in good agreement with the results of the analysis carried out by Zobetz (1982 ▸) on 225 B—O distances belonging to 75 BO_3_ groups [1.370 (19) Å]. Addison *et al.* (1984 ▸) have proposed the parameter *τ*_5_ to distinguish whether a five-coordinated atom is in a trigonal–bipyramidal or a square-pyramidal environment. With *τ*_5_ = −0.01667*α* + 0.01667*β* = 0, where *β* > *α* are the two largest valence angles of the coordination polyhedron, namely *α* = O1—Co1—O3^i^ = 157.75° and *β* = O^i^—Co1—O3 = 157.75° [symmetry code: (i) *x*, −*y* + 1, *z*], a square-pyramidal environment is realized for the Co^2+^ cation in the structure of the title compound. Each of the two barium cations is surrounded by nine oxygen atoms forming distorted polyhedra with average distances for Ba1—O and Ba2–O of 2.791 and 2.891 Å, respectively.

## Comparison with related structures   

Comparison of the crystal structure of the title compound with those of other orthoborates with formula type *A*_2_ *M*(BO_3_)_2_ listed in Table 1 ▸ reveals that the first three compounds crystallize in the monoclinic system with the same space group (*C*2/*m*) but a halved unit-cell volume. α-Sr_2_Cu(BO_3_)_2_ and Pb_2_Cu(BO_3_)_2_ also crystallize in the monoclinic system but in space group *P*2_1_/*c*. The remaining compounds adopt an ortho­rhom­bic structure, except for the last, Ba_2_Mg(BO_3_)_2_, which is hexa­gonal. In the crystal structures of all these borates, the small metal *M* has either a coordination number of four (CuO_4_, ZnO_4_) or six (CuO_6_, MgO_6_, CaO_6_, CdO_6_). Accordingly, it is important to note the originality of the title structure with its five-coordination of the cobalt cation instead of four- or six-coordination for *M* in the other *A*_2_ *M*(BO_3_)_2_ compounds. Moreover, the linkage of [CoO_5_] polyhedra and one of the two (BO_3_)^3–^ anions by sharing an edge is different from other *A*_2_ *M*(BO_3_)_2_ structures where [*M*O_4_] or [*M*O_6_] polyhedra are linked to the (BO_3_)^3–^ anions only through their vertices.

## Synthesis and crystallization   

Single crystals of Ba_2_Co(BO_3_)_2_ were isolated from the melt, starting from a mixture of Ba(NO_3_)_2_, Co(NO_3_)_2_·6H_2_O and H_3_BO_3_ in a molar ratio of 2:1:2. The mixture was subjected to successive heat treatments at 673 K and at 1073 K. The obtained powder was melted at a temperature of 1433 K, followed by a slow cooling. The resulting product consisted of pink crystals corresponding to the title compound.

## Refinement   

Crystal data, data collection and structure refinement details are summarized in Table 3 ▸. The maximum and minimum remaining electron density peaks are at 0.47 Å from Ba1 and 1.08 Å from Co1, respectively.

## Supplementary Material

Crystal structure: contains datablock(s) I. DOI: 10.1107/S2056989019002597/wm5486sup1.cif

Structure factors: contains datablock(s) I. DOI: 10.1107/S2056989019002597/wm5486Isup2.hkl

CCDC reference: 1898300

Additional supporting information:  crystallographic information; 3D view; checkCIF report

## Figures and Tables

**Figure 5:**
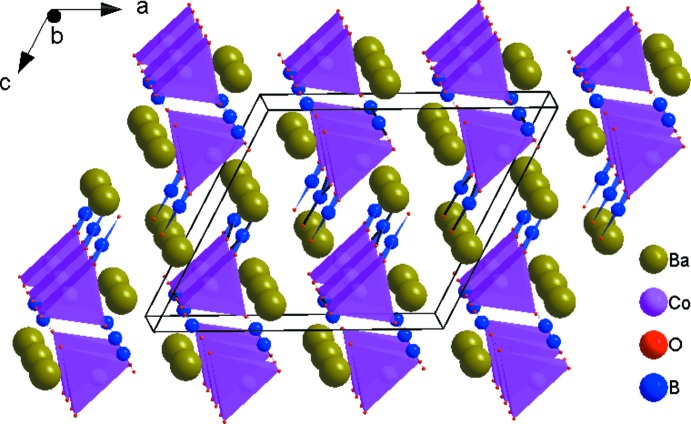
Projection of the crystal structure of Ba_2_Co(BO_3_)_2_ approximately along [010], emphasizing the voids between the cobalt borate rows in which the barium cations are located.

**Table 1:** Lattice parameters (Å, °), space groups and references for *A*_2_ *M*(BO_3_)_2_ compounds

Formula	*a*	*b*	*c*	β	*Z*	Space group	Reference
Ba_2_Ca(BO_3_)_2_	9.362 (2)	5.432 (2)	6.635 (2)	119.38 (1)	2	*C*2/*m*	Akella & Keszler (1995 ▸)
Ba_2_Cd(BO_3_)_2_	9.6305 (4)	5.3626 (3)	6.5236 (2)	118.079 (3)	2	*C*2/*m*	Zhang *et al.* (2011*b* ▸)
Sr_2_Mg(BO_3_)_2_	9.046 (4)	5.1579 (9)	6.103 (3)	118.691	2	*C*2/*m*	Chen *et al.* (2007 ▸)
Ba_2_Co(BO_3_)_2_	11.9784 (4)	5.3256 (2)	10.3220 (3)	117.494 (1)	4	*C*2/*m*	This work
α-Sr_2_Cu(BO_3_)_2_	5.707 (1)	8.796 (2)	6.027 (1)	116.98	2	*P*2_1_/*c*	Smith & Keszler (1989 ▸)
Pb_2_Cu(BO_3_)_2_	5.6311 (6)	8.7628 (9)	6.2025 (6)	115.706 (1)	2	*P*2_1_/*c*	Pan *et al.* (2006 ▸)
Ba_2_Cu(BO_3_)_2_	8.023 (1)	11.290 (1)	13.889 (1)		8	*Pnma*	Smith & Keszler (1990 ▸)
β-Sr_2_Cu(BO_3_)_2_	7.612 (3)	10.854 (7)	13.503 (4)		8	*Pnma*	Smith & Keszler (1989 ▸)
Ba_2_Zn(BO_3_)_2_	15.068 (2)	8.720 (2)	10.128 (3)		8	*Pca*2_1_	Smith & Koliha (1994 ▸)
Ba_2_Mg(BO_3_)_2_	5.343 (2)	5.343 (2)	16.520 (3)		3	*R* 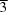 *m*	Kokh *et al.* (2017 ▸)

**Table 2:** Selected geometric parameters (Å, °)

Ba1—O3	2.7232 (12)	Co1—O2^iv^	2.0152 (18)
Ba1—O4	2.7266 (4)	Co1—O1	2.0432 (11)
Ba1—O1^i^	2.7697 (11)	Co1—O3	2.1043 (12)
Ba1—O4^ii^	2.8215 (19)	B1—O2	1.364 (3)
Ba1—O3^i^	2.9272 (12)	B1—O1	1.3943 (16)
Ba2—O3	2.7688 (12)	B1—O1^v^	1.3943 (16)
Ba2—O1^iii^	2.8064 (11)	B2—O4	1.377 (3)
Ba2—O2^iv^	2.9009 (8)	B2—O3^vi^	1.3920 (17)
Ba2—O1^iv^	2.9668 (12)	B2—O3	1.3920 (17)
Ba2—O4^ii^	3.1360 (17)		
			
O2—B1—O1	120.42 (9)	O4—B2—O3^vi^	122.38 (9)
O2—B1—O1^v^	120.42 (9)	O4—B2—O3	122.38 (9)
O1—B1—O1^v^	119.16 (18)	O3^vi^—B2—O3	115.18 (19)

Symmetry codes: (i) 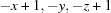; (ii) 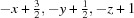; (iii) 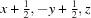; (iv) 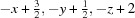; (v) 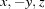; (vi) 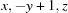.

**Table 3:** Experimental details

Crystal data	
Chemical formula	Ba_2_Co(BO_3_)_2_
*M*_r_	451.23
Crystal system, space group	Monoclinic, *C*2/*m*
Temperature (K)	296
*a*, *b*, *c* (Å)	11.9784 (4), 5.3256 (2), 10.3220 (3)
β (°)	117.494 (1)
*V* (Å^3^)	584.10 (3)
*Z*	4
Radiation type	Mo *K*α
μ (mm^−1^)	16.11
Crystal size (mm)	0.36 × 0.27 × 0.20

Data collection	
Diffractometer	Bruker D8 VENTURE Super DUO
Absorption correction	Multi-scan (*SADABS*; Krause *et al.*, 2015 ▸)
*T*_min_, *T*_max_	0.638, 0.746
No. of measured, independent and observed [*I* > 2σ(*I*)] reflections	12996, 1392, 1391
*R*_int_	0.032
(sin θ/λ)_max_ (Å^−1^)	0.806

Refinement	
*R*[*F* ^2^ > 2σ(*F* ^2^)], *wR*(*F* ^2^), *S*	0.015, 0.031, 1.41
No. of reflections	1392
No. of parameters	62
Δρ_max_, Δρ_min_ (e Å^−3^)	1.73, −1.12

Computer programs: *APEX3* and *SAINT* (Bruker, 2016 ▸), *SAINT*, *SHELXT2014/5* (Sheldrick, 2015*a* ▸), *SHELXL2016/6* (Sheldrick, 2015*b* ▸), *ORTEP-3 for Windows* (Farrugia, 2012 ▸), *DIAMOND* (Brandenburg, 2006 ▸) and *publCIF* (Westrip, 2010 ▸).

## supplementary crystallographic information

### Crystal data

**Table:**

Ba_2_Co(BO_3_)_2_	*F*(000) = 788
*M**_r_* = 451.23	*D*_x_ = 5.131 Mg m^−^^3^
Monoclinic, *C*2/*m*	Mo *K*α radiation, λ = 0.71073 Å
*a* = 11.9784 (4) Å	Cell parameters from 1392 reflections
*b* = 5.3256 (2) Å	θ = 3.4–35.0°
*c* = 10.3220 (3) Å	µ = 16.11 mm^−^^1^
β = 117.494 (1)°	*T* = 296 K
*V* = 584.10 (3) Å^3^	Block, colourless
*Z* = 4	0.36 × 0.27 × 0.20 mm

### Data collection

**Table:**

Bruker D8 VENTURE Super DUO diffractometer	1392 independent reflections
Radiation source: INCOATEC IµS micro-focus source	1391 reflections with *I* > 2σ(*I*)
HELIOS mirror optics monochromator	*R*_int_ = 0.032
Detector resolution: 10.4167 pixels mm^-1^	θ_max_ = 35.0°, θ_min_ = 3.4°
φ and ω scans	*h* = −19→18
Absorption correction: multi-scan (SADABS; Krause *et al.*, 2015)	*k* = −8→8
*T*_min_ = 0.638, *T*_max_ = 0.746	*l* = −16→16
12996 measured reflections	

### Refinement

**Table:**

Refinement on *F*^2^	0 restraints
Least-squares matrix: full	*w* = 1/[σ^2^(*F*_o_^2^) + (0.004*P*)^2^ + 1.2571*P*] where *P* = (*F*_o_^2^ + 2*F*_c_^2^)/3
*R*[*F*^2^ > 2σ(*F*^2^)] = 0.015	(Δ/σ)_max_ = 0.001
*wR*(*F*^2^) = 0.031	Δρ_max_ = 1.73 e Å^−^^3^
*S* = 1.41	Δρ_min_ = −1.12 e Å^−^^3^
1392 reflections	Extinction correction: SHELXL-2016/6 (Sheldrick, 2015*b*), Fc^*^=kFc[1+0.001xFc^2^λ^3^/sin(2θ)]^-1/4^
62 parameters	Extinction coefficient: 0.0101 (3)

### Special details

**Table:**

Geometry. All esds (except the esd in the dihedral angle between two l.s. planes) are estimated using the full covariance matrix. The cell esds are taken into account individually in the estimation of esds in distances, angles and torsion angles; correlations between esds in cell parameters are only used when they are defined by crystal symmetry. An approximate (isotropic) treatment of cell esds is used for estimating esds involving l.s. planes.

### Fractional atomic coordinates and isotropic or equivalent isotropic displacement parameters (Å^2^)

**Table:**

	*x*	*y*	*z*	*U*_iso_*/*U*_eq_	
Ba1	0.59046 (2)	0.000000	0.39992 (2)	0.00733 (4)	
Ba2	0.82861 (2)	0.000000	0.84816 (2)	0.00846 (4)	
Co1	0.61032 (3)	0.500000	0.79447 (3)	0.00765 (6)	
B1	0.5939 (2)	0.000000	0.9373 (2)	0.0065 (3)	
B2	0.6356 (2)	0.500000	0.5684 (3)	0.0073 (3)	
O1	0.53712 (10)	0.2258 (2)	0.87137 (12)	0.00920 (18)	
O3	0.63351 (11)	0.2793 (2)	0.63947 (13)	0.01062 (18)	
O2	0.70464 (16)	0.000000	1.0636 (2)	0.0151 (3)	
O4	0.64458 (17)	0.500000	0.4400 (2)	0.0120 (3)	

### Atomic displacement parameters (Å^2^)

**Table:**

	*U*^11^	*U*^22^	*U*^33^	*U*^12^	*U*^13^	*U*^23^
Ba1	0.00848 (6)	0.00626 (6)	0.00619 (6)	0.000	0.00250 (4)	0.000
Ba2	0.00697 (6)	0.00742 (6)	0.00910 (6)	0.000	0.00210 (4)	0.000
Co1	0.01018 (11)	0.00530 (11)	0.00871 (12)	0.000	0.00542 (10)	0.000
B1	0.0076 (8)	0.0057 (8)	0.0067 (8)	0.000	0.0038 (7)	0.000
B2	0.0073 (8)	0.0066 (8)	0.0079 (8)	0.000	0.0034 (7)	0.000
O1	0.0105 (4)	0.0064 (4)	0.0101 (4)	0.0006 (3)	0.0043 (4)	0.0018 (3)
O3	0.0151 (5)	0.0069 (4)	0.0094 (4)	−0.0003 (4)	0.0052 (4)	0.0006 (3)
O2	0.0105 (6)	0.0134 (7)	0.0127 (7)	0.000	−0.0020 (6)	0.000
O4	0.0197 (7)	0.0079 (6)	0.0132 (7)	0.000	0.0118 (6)	0.000

### Geometric parameters (Å, º)

**Table:**

Ba1—O3	2.7232 (12)	Ba2—O1^viii^	2.9668 (12)
Ba1—O3^i^	2.7232 (12)	Ba2—O1^x^	2.9668 (11)
Ba1—O4^ii^	2.7266 (4)	Ba2—O4^v^	3.1360 (17)
Ba1—O4	2.7266 (4)	Co1—O2^viii^	2.0152 (18)
Ba1—O1^iii^	2.7697 (11)	Co1—O1^xi^	2.0432 (11)
Ba1—O1^iv^	2.7697 (11)	Co1—O1	2.0432 (11)
Ba1—O4^v^	2.8215 (19)	Co1—O3^xi^	2.1043 (12)
Ba1—O3^iii^	2.9272 (12)	Co1—O3	2.1043 (12)
Ba1—O3^iv^	2.9272 (12)	B1—O2	1.364 (3)
Ba2—O3^i^	2.7688 (12)	B1—O1	1.3943 (16)
Ba2—O3	2.7688 (12)	B1—O1^i^	1.3943 (16)
Ba2—O1^vi^	2.8064 (11)	B2—O4	1.377 (3)
Ba2—O1^vii^	2.8064 (11)	B2—O3^xi^	1.3920 (17)
Ba2—O2^viii^	2.9009 (8)	B2—O3	1.3920 (17)
Ba2—O2^ix^	2.9009 (8)		
			
O3—Ba1—O3^i^	66.22 (5)	O1^vi^—Ba2—O2^viii^	75.01 (4)
O3—Ba1—O4^ii^	117.61 (5)	O1^vii^—Ba2—O2^viii^	134.17 (4)
O3^i^—Ba1—O4^ii^	52.88 (4)	O3^i^—Ba2—O2^ix^	64.04 (4)
O3—Ba1—O4	52.88 (4)	O3—Ba2—O2^ix^	123.27 (4)
O3^i^—Ba1—O4	117.61 (5)	O1^vi^—Ba2—O2^ix^	134.17 (4)
O4^ii^—Ba1—O4	155.16 (8)	O1^vii^—Ba2—O2^ix^	75.01 (4)
O3—Ba1—O1^iii^	160.05 (3)	O2^viii^—Ba2—O2^ix^	133.25 (7)
O3^i^—Ba1—O1^iii^	117.76 (3)	O3^i^—Ba2—O1^viii^	154.99 (3)
O4^ii^—Ba1—O1^iii^	73.21 (5)	O3—Ba2—O1^viii^	112.17 (3)
O4—Ba1—O1^iii^	123.99 (4)	O1^vi^—Ba2—O1^viii^	66.44 (4)
O3—Ba1—O1^iv^	117.76 (3)	O1^vii^—Ba2—O1^viii^	96.47 (3)
O3^i^—Ba1—O1^iv^	160.05 (3)	O2^viii^—Ba2—O1^viii^	48.15 (4)
O4^ii^—Ba1—O1^iv^	123.99 (4)	O2^ix^—Ba2—O1^viii^	103.68 (4)
O4—Ba1—O1^iv^	73.21 (5)	O3^i^—Ba2—O1^x^	112.17 (3)
O1^iii^—Ba1—O1^iv^	51.46 (5)	O3—Ba2—O1^x^	154.99 (3)
O3—Ba1—O4^v^	77.18 (4)	O1^vi^—Ba2—O1^x^	96.47 (3)
O3^i^—Ba1—O4^v^	77.18 (4)	O1^vii^—Ba2—O1^x^	66.44 (4)
O4^ii^—Ba1—O4^v^	77.69 (4)	O2^viii^—Ba2—O1^x^	103.68 (4)
O4—Ba1—O4^v^	77.69 (4)	O2^ix^—Ba2—O1^x^	48.15 (4)
O1^iii^—Ba1—O4^v^	122.58 (4)	O1^viii^—Ba2—O1^x^	58.98 (4)
O1^iv^—Ba1—O4^v^	122.58 (4)	O3^i^—Ba2—O4^v^	71.41 (4)
O3—Ba1—O3^iii^	100.43 (3)	O3—Ba2—O4^v^	71.41 (4)
O3^i^—Ba1—O3^iii^	68.02 (4)	O1^vi^—Ba2—O4^v^	66.68 (4)
O4^ii^—Ba1—O3^iii^	70.32 (4)	O1^vii^—Ba2—O4^v^	66.68 (4)
O4—Ba1—O3^iii^	130.98 (4)	O2^viii^—Ba2—O4^v^	112.77 (4)
O1^iii^—Ba1—O3^iii^	66.20 (3)	O2^ix^—Ba2—O4^v^	112.77 (4)
O1^iv^—Ba1—O3^iii^	92.16 (3)	O1^viii^—Ba2—O4^v^	132.76 (3)
O4^v^—Ba1—O3^iii^	142.41 (3)	O1^x^—Ba2—O4^v^	132.76 (3)
O3—Ba1—O3^iv^	68.02 (4)	O2^viii^—Co1—O1^xi^	104.04 (5)
O3^i^—Ba1—O3^iv^	100.43 (3)	O2^viii^—Co1—O1	104.04 (5)
O4^ii^—Ba1—O3^iv^	130.98 (4)	O1^xi^—Co1—O1	91.26 (6)
O4—Ba1—O3^iv^	70.32 (4)	O2^viii^—Co1—O3^xi^	93.78 (6)
O1^iii^—Ba1—O3^iv^	92.16 (3)	O1^xi^—Co1—O3^xi^	97.30 (4)
O1^iv^—Ba1—O3^iv^	66.20 (3)	O1—Co1—O3^xi^	157.75 (5)
O4^v^—Ba1—O3^iv^	142.41 (3)	O2^viii^—Co1—O3	93.78 (6)
O3^iii^—Ba1—O3^iv^	61.09 (5)	O1^xi^—Co1—O3	157.75 (5)
O3^i^—Ba2—O3	64.99 (5)	O1—Co1—O3	97.30 (4)
O3^i^—Ba2—O1^vi^	138.10 (4)	O3^xi^—Co1—O3	67.90 (6)
O3—Ba2—O1^vi^	100.67 (4)	O2—B1—O1	120.42 (9)
O3^i^—Ba2—O1^vii^	100.67 (3)	O2—B1—O1^i^	120.42 (9)
O3—Ba2—O1^vii^	138.10 (4)	O1—B1—O1^i^	119.16 (18)
O1^vi^—Ba2—O1^vii^	62.72 (5)	O4—B2—O3^xi^	122.38 (9)
O3^i^—Ba2—O2^viii^	123.27 (4)	O4—B2—O3	122.38 (9)
O3—Ba2—O2^viii^	64.04 (4)	O3^xi^—B2—O3	115.18 (19)

Symmetry codes: (i) *x*, −*y*, *z*; (ii) *x*, *y*−1, *z*; (iii) −*x*+1, −*y*, −*z*+1; (iv) −*x*+1, *y*, −*z*+1; (v) −*x*+3/2, −*y*+1/2, −*z*+1; (vi) *x*+1/2, −*y*+1/2, *z*; (vii) *x*+1/2, *y*−1/2, *z*; (viii) −*x*+3/2, −*y*+1/2, −*z*+2; (ix) −*x*+3/2, −*y*−1/2, −*z*+2; (x) −*x*+3/2, *y*−1/2, −*z*+2; (xi) *x*, −*y*+1, *z*.
